# Decreased hydration status of emergency department physicians and nurses by the end of their shift

**DOI:** 10.1186/1865-1380-6-27

**Published:** 2013-07-17

**Authors:** Mohammed Z Alomar, Abdullah Akkam, Samer Alashqar, Abdelmoneim Eldali

**Affiliations:** 1 Emergency Department, King Faisal Specialist Hospital and Research Centre, Altakhassusi, Riyadh 11211, Saudi Arabia; 2 Biostatistics Department, King Faisal Specialist Hospital and Research Centre, Altakhassusi, Riyadh 11211, Saudi Arabia

**Keywords:** Emergency, Shift, Physician, Nurse, Hydration

## Abstract

**Background:**

Typical emergency department (ED) shifts are physically demanding. The aim of this study was to assess the hydration status of ED physicians and nurses by the end of their shifts.

**Methods:**

A prospective cross-sectional clinical study of ED physicians and nurses assessing fluid intake, activities, vital signs, weight, urine specific gravity and ketones at the end of the shift. Forty-three participants were tested over 172 shifts distributed over 48% in the morning, 20% in the evening and 32% at night. Fifty-eight percent were females, and 51% were physicians.

**Results:**

Overall, participants lost 0.3% of their body weight by the end of the shift. While physicians lost a mean of 0.57 kg (± SD 0.28; *P* < 0.0001, 95% CI 0.16-0.28), nurses lost 0.12 kg (± SD 0.25; *P* < 0.0001, 95% CI 0.07-1.7). While nurses drank more fluid (*P* < 0.0001), physicians had a higher specific gravity of 1.025 (*P* < 0.01), visited the washroom less often (*P* < 0.0001) and reported less workload and stress (*P =* 0.01 and 0.008, respectively). There were no major changes in vital signs or urinary ketones (OR.0.41, 95% CI 0.1-2.1). In a multivariate analysis, being male (OR 13.5, 95% CI 1.6-112.5), being of younger age (OR 4.1, 95% CI 1.7-10.2), being Middle Eastern (OR 5.3, 95% CI 1.1-26.2), working the morning shift (OR 2.7, 95% CI 0.7-10.5) and having less fluid intake (OR 5.7, 95% CI 1.2-26.6) were significant predictors of decreased hydration.

**Conclusions:**

The majority of physicians and to a lesser extent nurses working in a tertiary care emergency department have decreased hydration status at the end of the shift. Therefore, awareness of the hydration status by emergency department staff is needed. A further study in a similar setting with more subjects and a better balance among the variables is recommended.

## Background

The typical emergency department (ED) shift is not only cognitively demanding, requiring complex decision making in a fast-paced environment, but is also physically demanding. During a regular shift, ED physicians and nurses may be far removed from areas that provide access to fluids and, when available, cannot keep it handy for infection control purposes. As a result, they are often unable to drink properly or at all during their shifts; especially in warm countries, this can lead to increased perspiration and higher water requirements. Equally, working in an air-conditioned environment, moisture evaporation speeds up, resulting in increased water loss and the need to consume more fluids.

Previous studies have demonstrated that proper hydration plays a significant role in human performance and dehydration as low as 2% of the body weight has been shown to adversely influence decision-making and cognitive performance, which may contribute to a decline in productivity and could be associated with an increased risk of work-related accidents [1-14].

Gopinthan et al. [15] dehydrated subjects passively by 1, 2, 3 and 4% of body weight and found that visual motor tracking, short-term memory, attention and arithmetic efficiency were all impaired at 2% or more dehydration. Szinnai et al. [16] examined the effect of 2.6% dehydration on the cognitive-motor function of 16 subjects and found that dehydrated subjects reported greater tiredness, reduced alertness, and higher levels of perceived effort and concentration compared to their normally hydrated state.

A previous study by Verbalis [17] showed that the brain is particularly sensitive to changes in water balance, which in turn can affect mental performance (e.g., concentration, alertness and short-term memory) [18] and overall work-related productivity.

Cian et al. [19] observed the effect of 3% dehydration achieved by exercise or heat exposure on cognitive function. They reported that, compared to being in a well-hydrated state, dehydrated subjects exhibited impairments in short-term memory and reported greater fatigue for up to 2 h following dehydration.

Physicians and nurses are often unable to drink properly during their shift, and we feel they are not hydrated well enough. However, there is a lack of research empirically examining the hydration status and its effect on ED staff during their shifts. Good hydration may ultimately benefit not only the physicians and nurses themselves, but also their patients and the health-care systems in which they work [15,16].

We aim to assess the hydration status of ED physicians and nurses by the end of their shifts.

## Methods

### Aim and hypothesis

We hypothesized that emergency department physicians and nurses should not be dehydrated by the end of their working shifts. Hence, the aim of this study was to examine the hydration status of ED physicians and nurses by the end of their shifts.

### Design

This was a prospective cross-sectional clinical study of ED physicians and nurses assessing their hydration status during their shifts. Following a department-wide poster campaign advertising the study recruitment location and timing, physicians and nurses with similar shifts (days, evenings and nights) were recruited.

### Information and consenting process

Physicians and nurses were informed about the study ahead of time. They were informed that the study was going to assess their hydration status at the end of every shift by asking questions about the participants’ activities and fluid intake before and by the end of their shift. Later on, weight, vital signs, urine dip for ketones and specific gravity (SpG) values would be checked. A research participant information sheet was distributed to all participants. Informed consent was taken in writing by the principal investigator at the beginning of the study.

### Study subjects /setting

The Department of Emergency Medicine at King Faisal Specialist Hospital and Research Centre (KFSHRC) is a training area for many residency and fellowship training programs. The annual number of ED visits is about 65,000 patients per year with around 25% being <14 years old. There are 32 beds including 2 for resuscitation, and it covers about 500 m^2^. The average temperature in the ED is 24–25 C° all year around. There are 76 registered nurses, and 14 adult emergency medicine (EM) and 12 pediatric emergency medicine (PEM) consultants. The average number of residents and fellows rotating monthly is ten.

**Figure 1:**
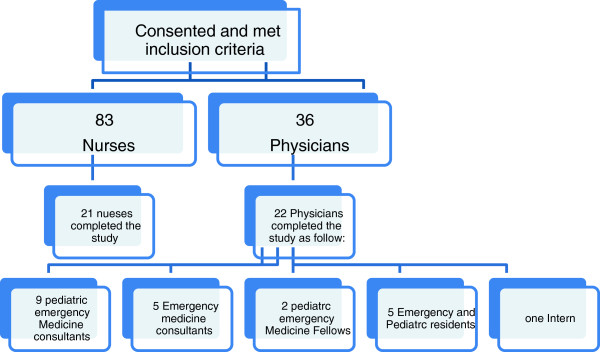
The population of physicians and nurses recruited for the study on dehydration.

Typical shifts for physicians including the trainees are morning (08:00–16:00), afternoon (16:00–24:00) and night shifts (00:00–08:00). Nurses’ shifts are morning (07:00–19:00) and night (19:00–07:00). The nurses were tested 8 h after the beginning of their shift in order to match up with the physicians’ 8-h shift. In this case, the nurses’ morning and night shift replaced some of the afternoon shifts. The study target was all physicians and nurses working in the ED (Figure 1).

Physicians and nurses not willing to participate or taking diuretics or any other medications affecting their hydration status were excluded.

### Data collection and analysis

1. *Questionnaires*

Questionnaires including baseline demographic characteristics, physical activity and fluid intake were recorded at study enrollment at the beginning and at the end of every shift; participants were weighed, vital signs taken and urine tested. On the study shift, participants maintained their usual eating and drinking habits.

The measure of weight, observed by the investigators at the beginning and end of each study shift, was standardized by using a single digital scale at the same location and ensuring participants’ equivalent post urinary void state and clothing status (e.g., shoes off, pockets empty, pagers removed). Participants were asked to rate both shifts on scales of 0 (none) to 10 (high) for workload, stress and general wellbeing. The physical status before the shift, amount of fluid taken prior to the shift (one cup was estimated to be 250 ml), number of cups of fluid and number of visits to the bathroom were charted.

2. *Physical measurements*

Weight and vital signs including heart rate, respiratory rate, blood pressure and pulse oximetry were taken in the first and last hour of the shift.

3. *Biochemical measures*

Urine tests including dipping for ketones and specific gravity were taken in the first and last hour of the shift.

### Sample size

We set the type 1 error rate at a low level (alpha = 1%) to account for the multiplicity of testing, with a power of 95%. Moreover, according to the literature we took a correlation between responses across shifts that is assumed constant and set at r = 0.15. To detect a clinically meaningful difference = 0.12 KG, we needed 169 shifts.

### Outcomes

The primary outcome was a change in weight and/or urine specific gravity. The secondary outcome was the change related to staff rank, age, type of shift, stress and workload.

### Statistical analysis

Demographic characteristics of subjects were presented as percentages for categorical variables and as mean ± SD for continuous variables. The effect of shift was assessed using repeated measures ANOVA. To account for possible sources of variation among subjects (e.g., age, gender, ethnicity. etc.), we used the linear mixed regression model. We set the type 1 error at 5%.

A repeated-measures ANOVA design was used to test for significant differences (*P* < 0.05) between measurement at the two stages, i.e., at baseline and the end of the shift. Dependent variables included weight, vital signs and urine specific gravity.

For the present investigation, the “condition” was hydration status; the presence of the condition was dehydration, and absence of the condition was euhydration. The “diagnostic tests” evaluated were weight loss of ≥1% and/or major changes in vital signs supported by positive ketones in urine and/or urine-specific gravity at a designated cutoff of 1.020; a test value <1.020 represents a negative test and a test ≥1.020 a positive test [20].

### Study duration

The emergency physician and nurse candidates were tested over the 6 months from the beginning of November 2011 till the end of April 2012.

### Ethics

The confidentially of collected data was protected, and Office of Research Affairs approval was obtained.

## Results

Forty-three participants were tested over 172 shifts distributed over 48% in the morning, 20% in the evening and 32% at night (Figure 1). The reasons for those who did not complete the study were time constraints, rotation in other areas (trainees) and lack of incentives. The majority were females (58%). The higher female ratio was explained by the higher ratio of females in the nursing staff. Table 1 shows the demographic characteristics of the participants.

**Table 1:** Demographic characteristic of physicians and nurses participating in the hydration study

**Rank**		**Number (%)**
Nurses		84 (49)
Physicians (88)	Interns	4 (2.3)
	Residents	20 (11.6)
	PEM fellows	8 (4.65)
	PEM consultants	36 (20.9)
	EM consultants	20 (11.6)
Gender	Male	72 (41.9)
	Female	100 (58.1)
Age (years)	20-30	56 (32.6)
	31-40	36 (20.9)
	41-50	52 (30.2)
	51-60	20 (11.6)
	Over 61	8 (4.7)
Race	Caucasian	28 (16.3)
	African	4 (2.3)
	Asian	64 (37.2)
	Middle Eastern	76 (44.2)
	Caucasian	28 (16.3)
Type of shift	Morning	82 (47.7)
	Afternoon	34 (19.8)
	Night	56 (32.6)3

**Figure 2:**
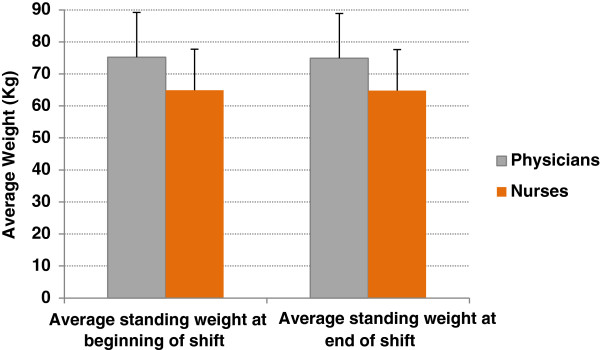
Differences between the average standing weight (kg) at the beginning and end of shifts between physicians and nurses.

**Figure 3:**
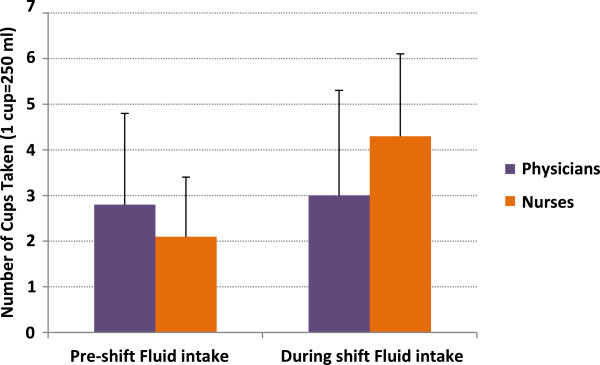
Total amounts of fluids taken before and during the shift.

**Figure 4:**
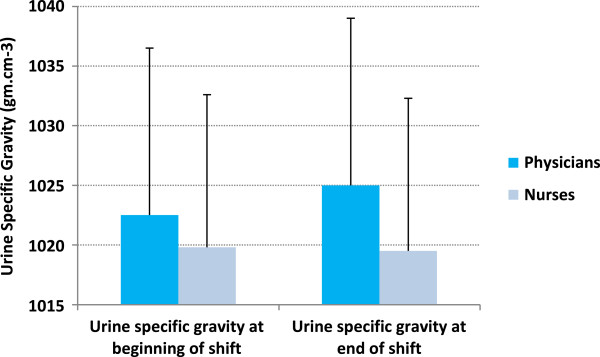
Urine specific gravity of participants at the beginning and end of the shift.

**Figure 5:**
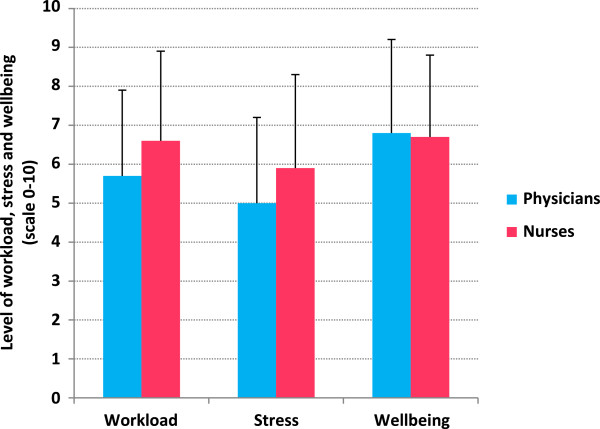
Workload, stress and wellbeing level of the participants at the end of the shift.

The average age for physicians was bimodally 20–30 years and 40–50 years, while for nurses it was 20–30 years. Overall, participants lost 0.3% of their body weight by the end of their shifts. While physicians lost a mean of 0.57 kg (± SD 0.28; *P* < 0.0001, 95% CI 0.16-0.28), nurses lost 0.12 kg (± SD 0.25; *P* < 0.0001, 95% CI 0.07-1.7) (Figure 2). While nurses drank more fluid (*P* < 0.0001) (Figure 3), physicians had higher mean specific gravity of 1.025 (*P* < 0.01) (Figure 4), visited the washroom less often (*P* < 0.0001) and reported less workload and stress (*P =* 0.01 and 0.008, respectively) (Figure 5). There were no major changes in vital signs or urinary ketones (OR.0.41, 95% CI 0.1-2.1).

In a multivariate analysis, being male, having younger age, being Middle Eastern, working the morning shift and less fluid intake were significant predictors of dehydration (Table 2).

**Table 2:** Predictors of decreased hydration of physicians and nurses during their shifts

**Variable**	**Adjusted odds ratio**	***P value***
	**(95% CI)**	
Male	3.6 (1.9-6.5)	<0.05
Younger age	2.1 (1.1-4.0)	<0.05
Middle Eastern	1.9 (1.1-3.4)	<0.05
Morning shift	2.2 (1.2-3.9)	<0.05
Less fluid intake	1.7 (1.0-3.0)	<0.05

## Discussion

To our knowledge, this is the first study done assessing the hydration status of emergency department physicians and nurses by the end of their work shifts. The inclusion of subjects with different races, different age groups and working in a shift-based environment makes this study unique. The study results showed that both physicians and nurses had become dehydrated by the ends of their shifts.

Assessing hydration states has proven challenging, given the difficulty in providing an accepted definition of normal body hydration status. Despite this difficulty, measuring total body water or plasma osmolality has been purported to be the standard for hydration assessment [21]. However, methods of measuring these variables such as isotopic dilution, bioelectrical impedance, and analysis of blood and/or plasma can be invasive, expensive and difficult to employ in a workplace setting. Urine specific gravity and ketones were used in this study as the sole biochemical markers. This method is less invasive but has limitations. Urine specific gravity has been shown to be somewhat effective in determining hydration status. Urine specific gravity of ≤1.020 was reported to be indicative of a normal hydration state and has been used to determine dehydration states in the workplace [22-25].

A urine specific gravity cutoff value of less than 1.020 was chosen in this study for euhydration, similar to the value used by the National Athletic Trainers Association (NATA) and Oppliger [26,27], but others have suggested this cutoff value might be too low [20,28-30].

The NATA associates minimal dehydration (1% to 3% of weight) with straw-colored urine and SpG of 1.010 to 1.020 [29]. Popowski et al. [31] monitored urine osmolality and SpG progressively as subjects dehydrated from baseline to 5% body weight. At 5% dehydration, the mean SpG was 1.021. Accordingly, physicians and nurses in this study were found to have mean specific gravities of 1.025 and 1.020 (Figure 4), which might be interpreted as dehydration of more than 5% and 3%, respectively.

Dehydration has been shown to adversely influence decision-making and cognitive performance, which may contribute to a decline in productivity and could be associated with an increased risk of work-related accidents, and both physicians and nurses in this study reported less wellbeing. Only physicians reported less workload and stress by the end of their shifts (Figure 5).

During physical work in the heat, sweat output can often exceed water intake, which can lead to body water deficits or dehydration. Bishop et al. [32] observed that, in simulated industrial work conditions, encapsulated protective clothing increased sweat rates up to 2.25 l/h. Likewise, wearing protective equipment such as full or half face masks as well as gowns by the study participants can make fluid consumption more difficult and can further contribute to dehydration. It is well documented that workers often not only become dehydrated on the job, but may also start the workday with a fluid deficit similar to our physicians.

Drinking fluids at regular intervals will help to replenish lost fluids before dehydration sets in, which is important because by the time a person actually feel thirsty, the body’s water level is likely to already be lower than it should be. Nurses and physicians consumed an average of 3–4 cups per 8-h shift (Figure 3). Work safety organizations, such as the UK Health and Safety Executive (HSE), state that in the workplace there should be adequate supplies of water, taking the temperature of the work environment and type of activity into consideration [33]. Equally, the US Occupational Safety and Health Administration (OSHA) advises that workers exposed to heat stress should drink fluids on a regular basis, e.g., around 250 ml (1 cup) of water every 20 min [34]. However, the majority of legislative guidelines provide vague guidance, and none take into account the effects of work intensity, specific environments or protective clothing. Health-care organizations might also falter in the provision of even basic resources for physician wellness and self-care, such as adequate rest, recovery and nutrition [35].

Improvement of physician wellness can improve the organization’s wellbeing and employee health, and physician wellness should receive the same priority as patient care and financial viability. That is, individual physician wellness is a valid indicator of organizational health [13,36]. While the physicians in this study had no breaks, nurses had a 1-h break per 12-h shift. Employers have sometimes not promoted drinking, as this would require more rest breaks and thus decrease employee productivity. It is more likely that sustaining hydration will maintain worker productivity sufficiently to offset any work breaks, particularly during hot weather. In addition, the decrease in health-care costs associated with possibly reducing accidents or illnesses in the workplace could further help the small decline in productivity from rest breaks. Using established meal breaks in a workplace setting, especially during longer work shifts (10 to 12 h), may help replenish fluids and can be important in replacing sodium and other electrolytes.

One recommendation that may enhance hydration at the work site involves improving access to bathroom facilities. Nurses visited the accessible bathrooms more often, in contrast to anecdotal statements and interviews that revealed that individuals, particularly women, will purposefully not drink fluids when bathroom facilities are not available. While logistical factors may complicate access to facilities, providing access may be a simple means of improving workplace hydration and reducing the practice of voluntary dehydration.

Education is a vital component to help workers maintain their hydration state during and after a work shift. Informing individuals, especially those who work in a warm environment, about hydration assessment, signs and dangers of dehydration, and strategies to maintain hydration while working can reduce dehydration in the workplace. Brake et al. [37] reported that individuals working in a thermally stressful environment were better able to maintain hydration when they were educated about dehydration, assessed their hydration state and used a fluid replacement program while working. An education and hydration program at work should stress the importance of consuming meals. De Castro [38] observed food and fluid intake of 36 adults over 7 consecutive days and concluded that the amount of fluid ingested was primarily related to the amount of food ingested and that fluid intake independent of eating was relatively rare. In addition, Maughan et al. [39], among others, reported that meals play an important role in helping to stimulate the thirst response, causing the intake of additional fluids and restoration of fluid balance.

The main limitation of this study was that the number of participants was low, which led to a wide margin of error and was reflected in the large variability in the error bars. This was a non-controlled prospective study. *P*-values were calculated by using independent Student’s *t*-test. The Satterthwaite method was used when there was variability in variances.

The use of the mixed linear model for the analysis, which is a general form of multiple regressions, allowed us to control for the possible resulting bias. We acknowledge that this was one of the limitations of the study; however, this issue could be dealt with using the new methodology of “propensity score matching.” This new methodology is quite technical, and using it in here might not have changed the final conclusions reported in this article.

A second limitation was the lack of either testing nurses by the end of their 12-h shift or testing physicians by the end of their 12-h shift, which is the practice in some other emergency departments. For further generalization of results, these variables would also need to be taken into account in future research. A third potential limitation was studying the shift load in detail to correlate the amount of work with the degree of dehydration. A fourth limitation was the lack of clinical outcomes or ED process measures, suggesting an effect of dehydration on performance. The fifth limitation was the lack of comparison with a control group. What would have happened if the same group had spent the same amount of time watching TV, playing a sport or going to work in another occupation? The sixth limitation was that although working the morning shift was a significant predictor of decreased hydration, which can be explained by the decreased pre-shift hydration status to start with, morning shifts are favored more heavily than other shifts and might skew the data, as hydration status changes throughout the day. Lastly, the subjects were not blinded to the purpose of the study and might have subconsciously changed their hydration and bathroom use habits during the study period. More subjects need to be recruited and better balance among the aforementioned variables obtained for future study.

## Conclusions

The majority of physicians and to a lesser extent nurses working in a tertiary care emergency department have decreased hydration status at the end of their shifts. Therefore, awareness of hydration status by emergency department staff is needed. Further study in a similar setting with more subjects and a better balance among the variables is recommended.

## Abbreviations

ED: Emergency department; SpG: Specific gravity; KFSHRC: King Faisal Specialist Hospital and Research Centre; EM: Emergency medicine; PEM: Pediatric emergency medicine; SD: Standard deviation; P: Probability value; CI: Confidence interval; NATA: National Athletic Trainers Association; HSE: Health and Safety Executive; OSHA: Occupational Safety and Health Administration.

## Competing interests

There are no financial or non-financial competing interests to disclose.

## Authors’ contributions

MA: Study concept and design, acquisition of the data, analysis and interpretation of the data, drafting of the manuscript, critical revision of the manuscript for important intellectual content and study supervision. AA and SA: Acquisition of the data. AE: Statistical expertise. All authors read and approved the final manuscript.
